# Enhancing Efficiency in Meniscal Repair: A Novel In-House Vacuum Suction Technique for Suture Loading in Long Hollow Needles and Devices

**DOI:** 10.7759/cureus.72775

**Published:** 2024-10-31

**Authors:** Srinivas B S Kambhampati, Mounika N S Chodavarapu, Harsha Bhogadi, B Skylab Naik, Sunil Kumar Koneru, Chinta Shyam Kumar, Riccardo D'Ambrosi

**Affiliations:** 1 Orthopaedics, Sri Dhaatri Orthopaedic, Maternity and Gynaecology Center, Vijayawada, IND; 2 Orthopaedics, Siddhartha Medical College, Vijayawada, IND; 3 Orthopaedics, Guntur Medical College, Guntur, IND; 4 Orthopaedics, Dr Pinnamaneni Siddhartha Institute of Medical Sciences and Research, Gannavaram, IND; 5 CASCO Department, Istituto di Ricovero e Cura a Carattere Scientifico (IRCCS) Istituto Ortopedico Galeazzi, Milan, ITA; 6 Biomedical Sciences for Health, UniversitÃ degli Studi di Milano, Milan, ITA

**Keywords:** long hollow needle, regular short needle, suture loading, unloaded suture anchor, vacuum suction

## Abstract

Background: Suture-loaded long hollow needles are used in arthroscopic surgery to pass or retrieve sutures and in meniscal repair surgeries. However, manual suture loading can be time-consuming and challenging, especially with finer sutures. We present a novel technique using vacuum suction to simplify and expedite suture loading in hollow needles and similar devices.

Methods: Following ethical approval and participant consent, four healthcare professionals (one senior consultant, two junior consultants, and one trained operating theatre technician) attempted to load 16G and 18G needles, as well as free and double-loaded commercial anchor drivers, using manual and vacuum suction techniques. The best of three attempts was recorded for each participant. Loading times were compared using permutation tests, and inter-operator variability was analyzed via F-tests.

Results: Across all devices, the suction method reduced loading times significantly compared to manual loading, with times dropping to 2-4 seconds. The senior consultant required 15 seconds for manual loading, while the untrained junior consultant took up to 60 seconds. Permutation tests revealed statistically significant time reductions with suction loading (p = 0.0286 for all devices). F-tests showed a substantial decrease in variability with suction loading, confirming greater consistency across users.

Conclusion: Vacuum suction is a quick, economical, and effective technique for loading sutures into long hollow needles and other devices. It not only drastically reduces loading times but also improves consistency across different users, making it a reliable technique for arthroscopic procedures. This method can potentially be applied to various surgical instruments, improving procedural efficiency and outcomes.

## Introduction

Suture-loaded long hollow needles are commonly used in arthroscopic surgery to deliver a loop of suture into the working area for passing or retrieving sutures from inside the knee joint. There are different approaches to delivering the loop of suture, including feeding the loop through a reversed Beath pin with the suture loaded into the eye of the needle or passing the loop using different types of suture passers from different companies. Procedures that use such retrieval of loops basically include procedures where tunnels are made to deliver or retrieve sutures from or inside the joint. These include repair of root tears of the meniscus, medialization of the meniscus, repair of avulsions of the tibial spine, etc. Suture-loaded needles are also regularly used in the outside-in repair [1] and inside-out repair [2] of meniscal tears, as well as loading sutures into the screwdriver used for a free, loaded suture anchor.

There are disadvantages to each type of suture loading that will be elaborated on in the discussion section. We have been using delivery of the loop using a long hollow needle since this needle has become part of our regular arthroscopy kit, as we use such needles for inside-out repair of meniscus tears [2]. For outside-in, also, the needles are short and frequently require manual loading of sutures. Currently, we load the transit suture (which is usually a size 2, 0, or size 2-0 fiber wire, or size 2 ethibond) into the needle manually.

Loading the suture into the needle takes time and is sometimes difficult due to the load compared to a stiffer suture because the thinner fiber wire of sizes 0 and 2-0 can be floppy and bend while loading, making it difficult to pass. It is relatively easier to pass a size 2 suture into a 16G needle due to the stiffness of the suture compared to the thinner sizes. We describe a technique for loading a suture into any long hollow needle effortlessly, and without fail, irrespective of how floppy or narrow the suture is. We compared manual loading to suction loading across different devices and personnel to give an idea of how easy or difficult each technique is. Such a technique has not been described previously in the literature.

## Materials and methods

**Figure 1:**
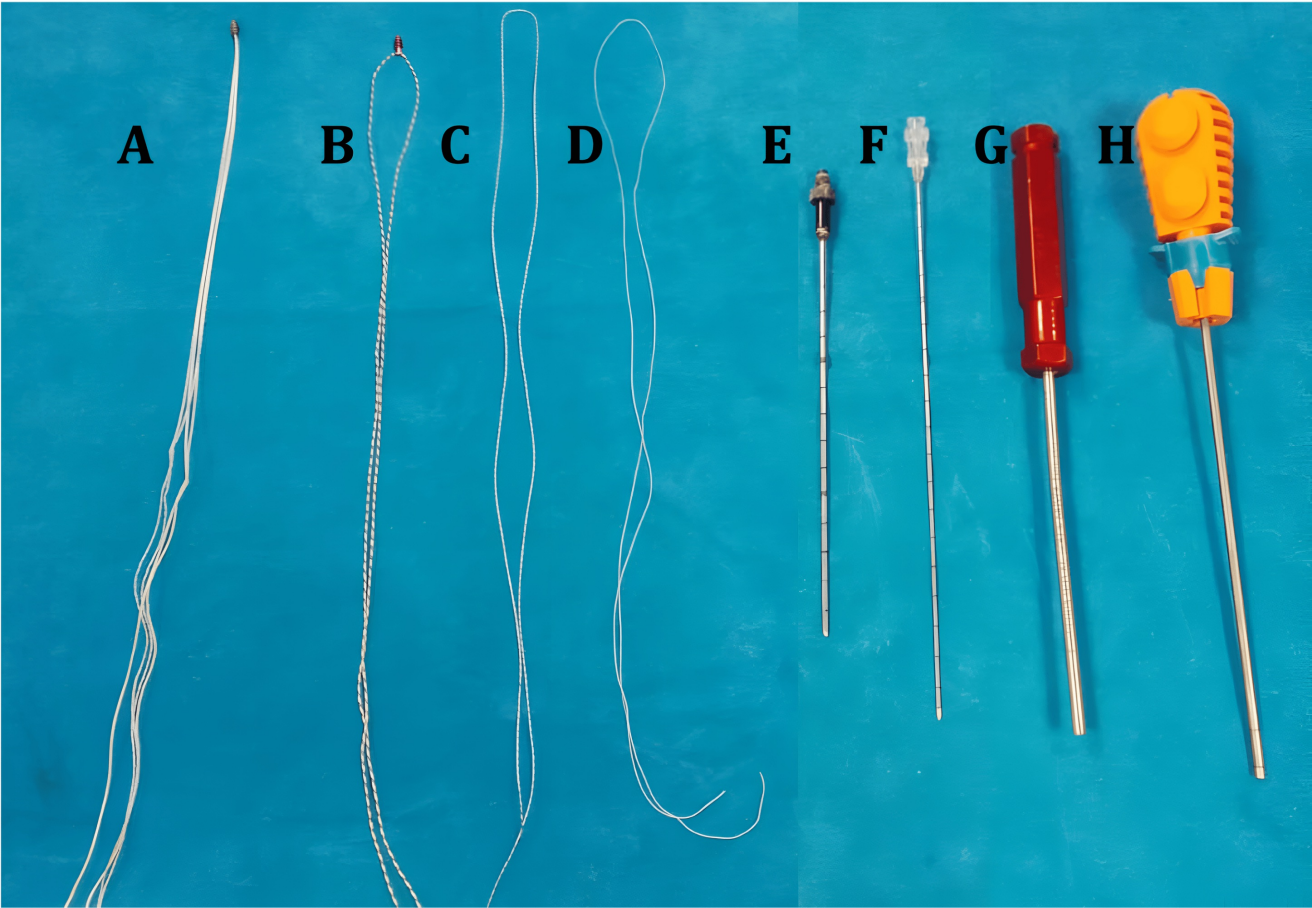
Materials used in this study. A: Double-loaded anchor with its suture, B: free anchor with its suture, C: size 0 fiber wire, D: 2-0 fiber wire, E: 16G needle 15 cm, F: 18G needle 20 cm, G: free single-loaded anchor deployer, and H: commercial double-loaded anchor deployer.

The basic requirements (Figure 1) are a regular vacuum suction with its tubing, which is commonly used in arthroscopic surgery to suck irrigation fluid, long hollow needles of 16G (15 cm) and 18G (20 cm) diameter, commercially available double-loaded anchor with its driver with the sutures taken out from the driver, and free anchor with single-loaded suture along with its driver and fiber wire sutures of sizes 0 and 2-0. Institutional ethical committee clearance was granted (IECSMCGGH/2024/AP/240). Consent was taken from all the participants of the study, and the study was conducted according to established norms.

A senior consultant, two junior consultants, and an operation theatre staff attempted to feed each of the sutures manually into different long hollow devices initially and was timed. The analysis was then based on the best times out of three attempts for each participant. Subsequently, the same attempts to feed were made using vacuum suction by them. The timings of each attempt were compared.

While performing with vacuum suction, one end of the suture was clamped with an artery forceps or asked to be held by an assistant to prevent the suture from getting sucked into the suction tubing, while the other end of the suture was fed into the sharp end of the hollow needle, leaving enough slack in the suture to be pulled into the needle at the other end, depending on the length of the needle.

**Figure 2:**
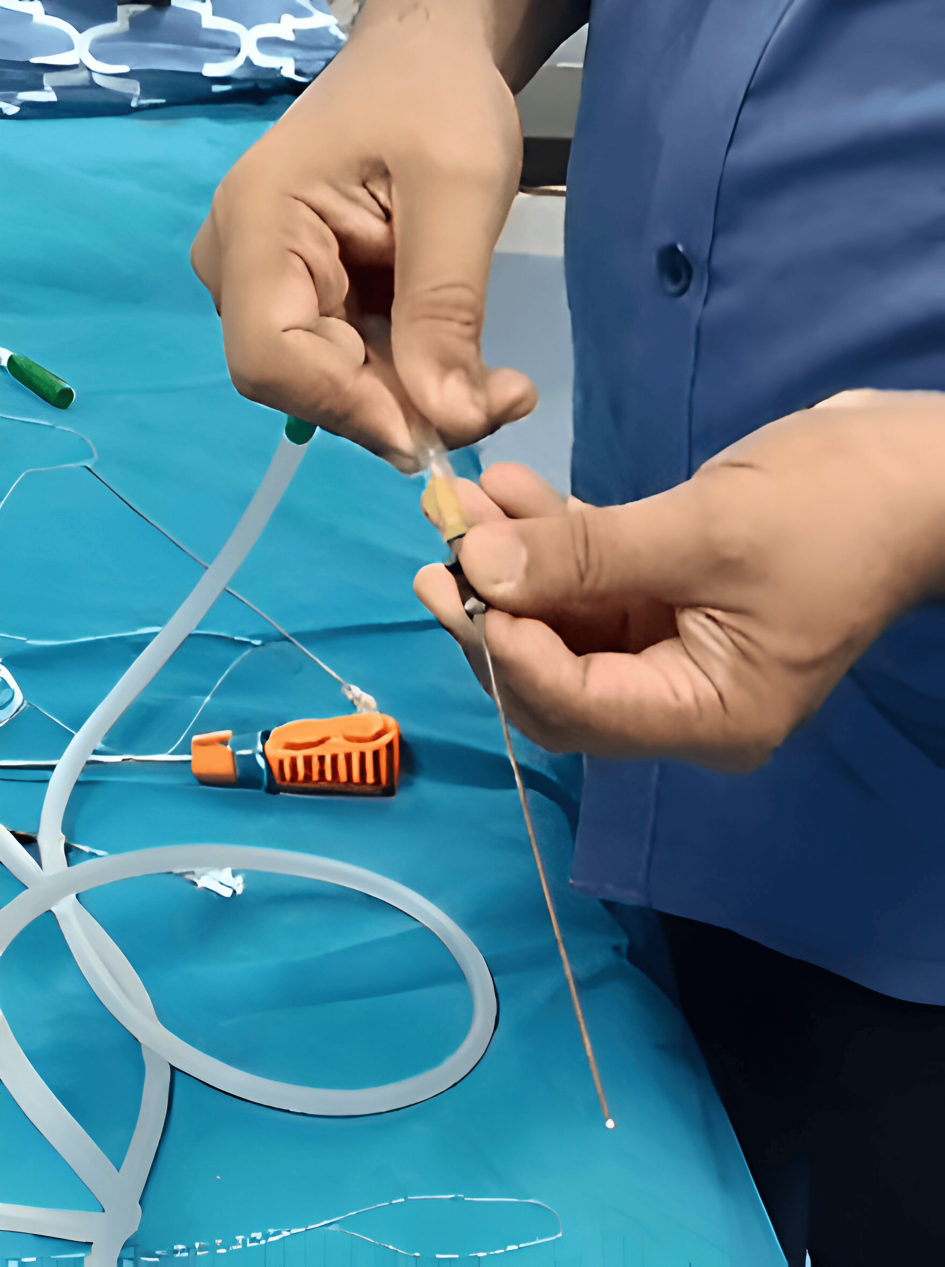
The connection of the suction device to a long biopsy needle. Commercial anchor deployer seen in the background.

Regular vacuum suction tubing is taken, and the nozzle of the suction is removed from the tubing. The tubing was then connected to the suction apparatus of a centralized vacuum suction mechanism (Figure 2). The hub of the needle usually fits into the hub of suction tubing for most long hollow needles. If required, additional short rubber tubing may be used to extend the hub to provide an airtight seal. The vacuum suction is turned off, and the hub of the needle is fed into the suction tubing, making sure the fit is airtight. After a satisfactory fit has been achieved, the suction is turned on, holding the suction side.

Once suction is turned on, the suture will be pulled into the needle. If the suture gets stuck, a gentle nudge or pulling back a bit will easily resolve the suture from the block, and the suture will be pulled into the needle, ending the free end of the suture in the tubing. The suture will be visible inside the tubing once it crosses the length of the needle. The suction is then turned off, and the hub end of the needle is disengaged from the tubing. The free end of the suture is now loaded and ready for use. The permutation tests suitable for small samples were performed to compare the mean times taken with and without suction for each device. The results were tabulated and compared.

## Results

**Figure 3:**
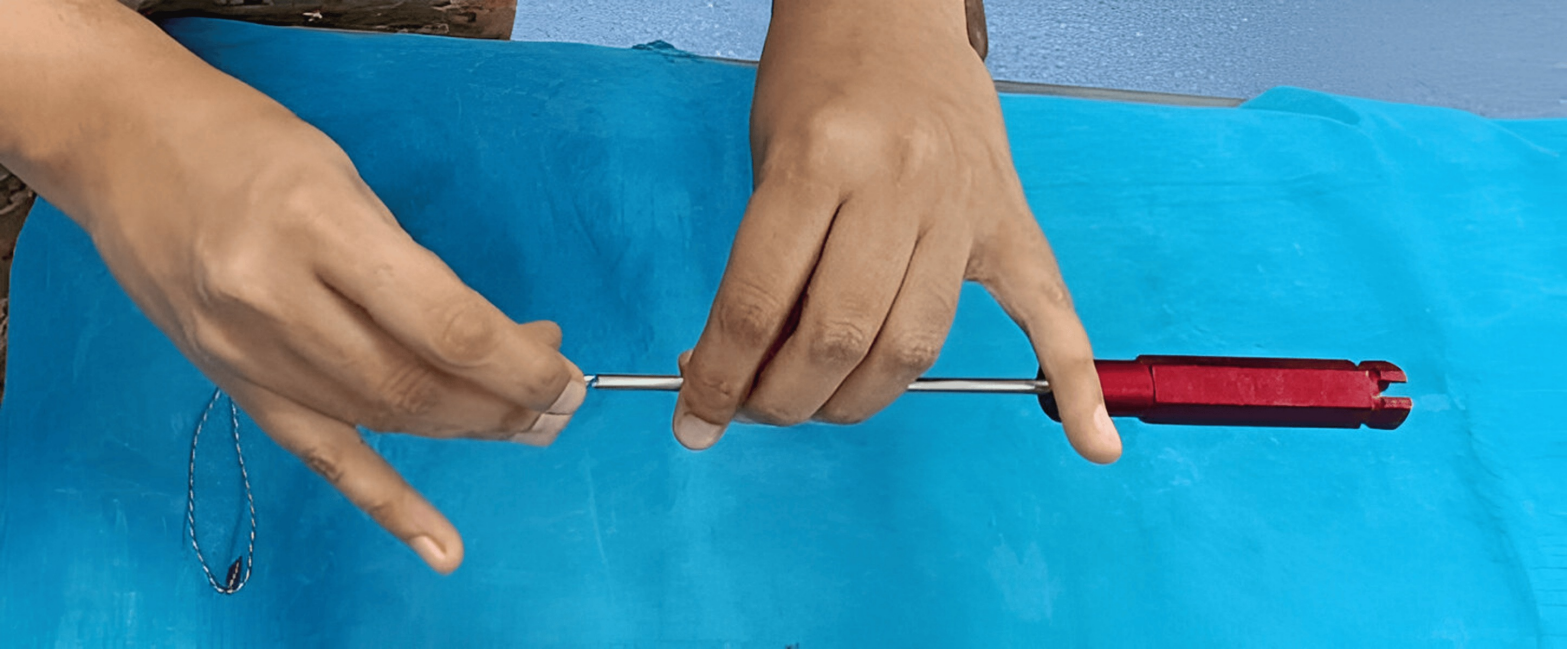
The manual feeding of a free anchor suture into an anchor driver.

**Figure 4:**
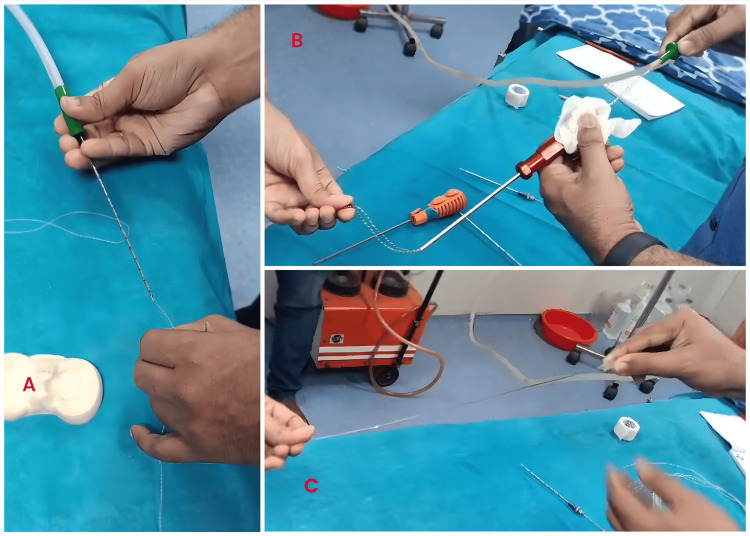
Loading suture into an 18G biopsy needle (A), free suture anchor (B) and to 16G needle (C) attached to a suction device with the suture, fiber wire and suture anchor being pulled into them due to the suction effect.

The results were tabulated in Table 1. While manual placement (Figure 3) took around 20 seconds for the senior consultant, the trained technician and a junior doctor took 24 and 21 seconds, respectively, and the untrained junior doctor took almost 60 seconds. With the suction tubing connected as described above, all four people could place the sutures into the needles in just 2 to 3 seconds (Figures 4A-4C). The trend was similar for other long hollow devices (Table 1).

**Table 1:** Loading times of hollow needles and anchor deployer without and with suction. OT: operation theatre.

Individual	16G needle		18G needle		Free anchor deployer		Commercial anchor deployer	
	Without suction (sec)	With suction (sec)	Without suction (sec)	With suction (sec)	Without suction (sec)	With suction (sec)	Without suction (sec)	With suction (sec)
Senior consultant	20	2	21	2	20	2	30	3
Trained OT technician	24	3	22	4	23	4	35	3
Trained junior consultant	21	2	22	2	22	2	30	3
Untrained junior consultant	60	3	45	3	50	4	60	4

Statistical analysis

The permutation tests were performed to compare the mean times taken with and without suction for each device, as non-parametric test does not assume normality and is ideal for small sample sizes and datasets that may not follow a normal distribution. P-values show statistically significant reductions in time with suction loading compared to manual loading (Table 2). This test shows that the time reduction is significant at the 0.05 level which confirms that the suction method is indeed faster and more efficient than the manual method.

**Table 2:** Permutation tests and mean times for the materials used in the study.

Device used	P-value	P-value significance	Mean time without suction (in seconds)	Mean time with suction (in seconds)
16 G needle	0.0286	Significant	31.25	2.5
18 G needle	0.0286	Significant	27.5	2.75
Free anchor deployer	0.0286	Significant	28.75	3
Commercial anchor deployer	0.0286	Significant	38.75	3.25

The advantages are that it is robust and flexible, working well even with small samples as seen in this study. It tests the actual observed data by generating a distribution of possible outcomes through resampling, making it distribution-free. The disadvantage of the test is that it may be less familiar to some readers or reviewers, as it is less commonly used than the paired t-test in certain fields, although it is becoming more accepted.

Statistical analysis for the 16G needle, 18G needle, free anchor deployer, and commercial anchor deployer is summarized in Table 2. The time taken to load the suture using the suction method was significantly less in all the groups (p<0.05) except for 16G needle group. For the 16G group, the mean times with and without suction were 2.5 and 31.25 seconds, respectively. The calculated inter-operator variability (standard deviation) for each group (Table 3) is as follows: The standard deviation (SD) for the 16G needle without and with suction was 19.24 seconds and 0.58 seconds, respectively, and the SD for the 18G needle without and with suction was 11.68 seconds and 0.96 seconds, respectively; similarly, for free anchor deployer: 1.15 seconds (with suction) vs 14.22 seconds (without suction) and for commercial anchor deployer: 0.50 seconds (with suction) vs 14.36 seconds (without suction).

**Table 3:** Standard deviation (inter-operator variability) without and with suction for all devices.

	SD without suction	SD with suction
16G needle	19.24 sec	0.58 sec
18G needle	11.68 sec	0.96 sec
Free anchor deployer	14.22 sec	1.15sec
Commercial anchor deployer	14.36 sec	0.50 sec

The statistical analysis of the data provided for different surgical tools used in suture loading revealed significant difference in the variances between methods with and without suction. The F-statistics calculated for each device were very high (Table 4), indicating substantial variability reduction when suction was applied compared to manual loading. For the 16G Needle, the F-statistic was 1110.75 with a P-value of 0.0000458, suggesting an extremely significant reduction in variability with suction. For the 18G needle, the F-statistic was 148.73 with a P-value of 0.000925, indicating significant improvements in consistency with the suction method. For the free anchor deployer, the F-statistic was 151.69 and the P-value was 0.000898, indicating a highly significant reduction in variability. Lastly, the commercial anchor deployer had a F-statistic of 825.00 and a P-value of 0.0000715, further supporting the substantial benefits of using suction. These results show that the differences in variability between manual and vacuum suction loading methods are statistically significant for all devices. These results show that the differences in variability between manual and vacuum suction loading methods are statistically significant for all devices.

**Table 4:** F-statistics and P-values.

Device category	F-statistic	P-value
16G needle	1110.75	0.0000458
18G needle	148.73	0.000925
Free anchor deployer	151.69	0.000898
Commercial anchor deployer	825.00	0.0000715

## Discussion

The main finding of this study is that vacuum suction reduces the loading times of fiber wires from five- to up to 30-fold for untrained personnel and reduces valuable operating time. Fiber wires and ethibond are used day in and day out in arthroscopic repair surgeries such as rotator cuff repair and meniscus repair by outside-in, inside-out, or all inside techniques, either through needles or with anchors. Loading sutures in the above cases is necessary either into free anchors or occasionally into double-loaded suture anchors [3], or long hollow needles or short regular needles for vertical or horizontal mattress suture placement in meniscal tears.

Permutation tests [4] are better suited to small samples where normality is in doubt, and they handle the magnitude of differences effectively, which is seen in this study with large reductions in time. Given the small sample size (n=4), we selected the permutation test as a robust non-parametric alternative to the paired t-test. The permutation test is particularly well-suited for small datasets as it makes no assumptions about the underlying distribution of the data, unlike the paired t-test, which assumes normality in the differences between paired measurements. The permutation test provides a P-value that reflects the likelihood of the observed difference occurring by chance. This makes it a flexible and robust test for detecting differences in paired data, especially when the sample size is limited.

Our technique significantly reduces the loading time of the suture into long hollow needles (Table 1), making it less than 5 seconds after connecting to the suction, while manual loading of the suture takes about 20 seconds to a minute to load into a 15- to 20-cm-long needle. A P-value of 0.0286 for all devices (Table 2) indicates that the reduction in time with suction loading is statistically significant. In other words, the time savings seen with the suction method are unlikely to have occurred by chance, and we can confidently reject the null hypothesis for this test. This test shows that the time reduction is significant at the 0.05 level, meaning that the suction method is indeed faster and more efficient than the manual method.

The longer the needle, the longer the manual loading takes, while suction loading takes almost negligible time to load into 15- and 20-cm-long needles. The most important step in our technique is to create an airtight closure between the suction nozzle and the needle or the anchor driver. If a gap appears in the connection, part of a free rubber glove may be used to fill in as a seal to create an airtight connection. We recommend using the wall-mounted central suction device as compared to a portable suction apparatus since the strength of suction using the portable device may not be satisfactory, especially for use with larger-diameter devices like the anchor deployers. Our technique is a quick way to load the suture into the needle. Multiple sutures may be loaded in one go, as in the case of a double-loaded anchor deployer, where four strands were suctioned simultaneously. Manual loading of the needle is fraught with delays, frustration from multiple attempts, and sometimes failure to load the suture due to the supple suture bending around the tip, especially a 2-0 suture.

Short hollow needles [1,5] and longer hollow needles [2] have been used for passing the suture for multiple arthroscopic procedures. These procedures include outside-in meniscal repair, inside-out meniscal repair [2], root repair of the meniscus using the tibial tunnel pull-through technique, and medialization of the extruded meniscus using the tibial tunnel pull-through technique. Any long hollow instrument used with a loaded suture may be loaded using our reported technique.

Free anchors, which are autoclavable and can be loaded during the procedure (Figure 4B) are connected to a hollow screwdriver for the deployment of the anchor. This anchor driver can be loaded with our technique, provided that an airtight seal is achieved. Even the regular packaged anchors, if the fiber wires require reloading into the anchor driver, for some reason, may be reloaded with all four sutures in a double-loaded anchor and two sutures in a single-loaded anchor. 18G needle requires a finer suture of size 2-0 or size 0 which is more supple compared to a size 2 suture which can fit into a 16G needle. Hence, loading the 18G needle is more difficult manually.

 We used the F-test to assess inter-operator variability between manual and suction methods. The F-test compares the variance of two groups and helps determine whether the variability in suture loading times was significantly reduced when using vacuum suction compared to manual loading. It was applied in our study to compare the consistency of performance between the two techniques among the different participants.

The F-test showed that loading using the suction method significantly reduced the variability when using the vacuum suction method compared to manual loading. The much lower standard deviations with suction (Table 3) indicate that this method is more consistent across different performers, reducing the influence of individual performer skill or technique.

For F-statistics and their P-values (Table 4), the results of the analysis unequivocally demonstrate the effectiveness of vacuum suction in reducing the time and variability associated with loading sutures into long hollow needles and anchor deployers. The use of vacuum suction leads to a statistically significant decrease in performance variability across different operators and tools, which is critical in high-stakes environments like surgical procedures. The following inferences can be made from the results given. The significant reduction in variability with suction suggests that this method provides a more consistent and reliable way of suture loading, which can be particularly beneficial in training settings and during complex surgeries where precision and time efficiency are paramount.

The decrease in time required to load sutures, as evidenced by lower mean times in the suction method, indicates enhanced operational efficiency. This can potentially lead to reduced operation times and increased throughput in surgical operations. Given the robustness of the results across different devices, the findings could have broader implications for various surgical procedures. Implementing vacuum suction could standardize more aspects of surgical operations, thereby potentially reducing the risk of errors and improving patient outcomes. These findings consistently support the hypothesis that using vacuum suction not only enhances the efficiency of suture loading but also significantly reduces the variability among operators, thereby improving overall procedural consistency.

The robustness of the F-test to non-normality, as detailed in the study by Blanca et al. [6], provides a strong foundation for its use in our analysis of the variability in suture loading times. This is particularly relevant given the practical scenarios in surgical settings, where data distributions often deviate from normality due to varied operator skill levels and procedural conditions. The authors’ extensive validation of the F-test across different non-normal distributions assures the reliability of our statistical conclusions, even under potential deviations from ideal statistical assumptions.

Haseler et al.'s [7] analysis of the impact of syringe size on vacuum generation and needle control provides a critical framework for understanding the broader implications of our findings. They highlighted that while larger syringes generated more vacuum, this often came at the cost of reduced control over the needle, which could lead to increased procedural complications. In our study, we leveraged vacuum suction technology to enhance control and precision in suture loading within arthroscopic surgeries, which is crucial for minimizing operational errors and improving patient outcomes.

In our vacuum suture suction system, employing finer needles under a consistent vacuum pressure head is theoretically advantageous. According to fluid dynamics principles [8], finer needles should induce higher suction velocities due to their reduced cross-sectional area, leading to more efficient and rapid suture loading. This enhanced suction efficiency could significantly improve the precision and speed of the suture loading process, which is critical in minimizing operational time and improving surgical outcomes.

A small, homogeneous sample of participants, primarily healthcare professionals, form a major limitation of our study. However, the personnel included are those who regularly perform orthopedic procedures with variable experiences and qualifications giving a broader spread. The study focuses primarily on the time saved by using the vacuum suction technique, with less emphasis on other important metrics such as the error rate, user satisfaction, and clinical outcomes following suture placement.

Broadening the study to include additional performance metrics such as error rates, complication rates, and overall user satisfaction could provide a more comprehensive analysis of the technique's impact. Clinical studies may be performed to evaluate usefulness during surgery. Future studies could include a larger and more diverse group of participants across multiple institutions.

## Conclusions

Our study conclusively demonstrates that vacuum suction suture loading not only simplifies the process of loading sutures into long hollow needles but also significantly reduces the time and effort involved, particularly for untrained personnel. This method consistently outperformed manual techniques, reducing suture loading times from up to a minute to just a few seconds, which is critical in high-stakes surgical environments where time and precision are of paramount importance. The vacuum suction technique not only speeds up the process but also makes it more reliable and reproducible across different users and does not rely on the individual skill set of the user.

This technique could be applied beyond arthroscopic surgery to any procedure requiring the use of long hollow needles, offering a versatile solution that can be implemented with minimal training and existing surgical infrastructure. This innovation represents a significant advancement in surgical efficiency and can potentially be adapted for broader medical applications.
